# Author Correction: Siliceous zeolite-derived topology of amorphous silica

**DOI:** 10.1038/s42004-025-01417-1

**Published:** 2025-01-28

**Authors:** Hirokazu Masai, Shinji Kohara, Toru Wakihara, Yuki Shibazaki, Yohei Onodera, Atsunobu Masuno, Sohei Sukenaga, Koji Ohara, Yuki Sakai, Julien Haines, Claire Levelut, Philippe Hébert, Aude Isambert, David A. Keen, Masaki Azuma

**Affiliations:** 1 Department of Materials and Chemistry, National Institute of Advanced Industrial Science and Technology, 1-8-31 Midorigaoka, Ikeda, Osaka 563-8577 Japan; 2 Center for Basic Research on Materials, National Institute for Materials Science, 1-2-1 Sengen, Tsukuba, Ibaraki 305-0047 Japan; 3 Institute of Engineering Innovation, The University of Tokyo, Yayoi 2-11-16, Bunkyo-ku, Tokyo 113-8656 Japan; 4 Photon Factory, Institute of Materials Structure Science, High Energy Accelerator Research Organization (KEK), 1-1 Oho, Tsukuba, Ibaraki 305-0801 Japan; 5 Institute for Integrated Radiation and Nuclear Science, Kyoto University, 2-1010 Asashiro-nishi, Kumatori-cho, Sennan-gun, Osaka 590-0494 Japan; 6 Graduate School of Engineering, Kyoto University, Kyotodaigaku-katsura, Nishikyo-ku, Kyoto 615-8520 Japan; 7 Institute of Multidisciplinary Research for Advanced Materials, Tohoku University, 2-1-1 Katahira, Aoba-ku, Sendai, Miyagi 980-8577 Japan; 8 Japan Synchrotron Radiation Research Institute (JASRI/SPring-8), Kouto, Sayo-cho, Hyogo 679-5198 Japan; 9 Kanagawa Institute of Industrial Science and Technology (KISTEC), 705-1 Shimoimaizumi, Ebina, Kanagawa 243-0435 Japan; 10 Laboratory for Materials and Structures, Tokyo Institute of Technology, 4259 Nagatsuta, Yokohama, Kanagawa 226-8503 Japan; 11 Institut Charles Gerhardt Montpellier, CNRS, Université de Montpellier, ENSCM, 34293 Cedex 5 Montpellier, France; 12 Laboratoire Charles Coulomb, CNRS, Université de Montpellier, 34095 Montpellier, France; 13 CEA, DAM Le Ripault, F-37260 Monts, France; 14 ISIS Facility, Rutherford Appleton Laboratory, Harwell Campus, Didcot, Oxfordshire OX11 0QX UK; 15 Present Address: Center for Basic Research on Materials, National Institute for Materials Science, 1-2-1 Sengen, Tsukuba, Ibaraki 305-0047 Japan; 16 Present Address: Faculty Materials for Energy, Shimane University, 1060 Nishikawatsu-cho, Matsue, Shimane 690-8504 Japan; 17 Present Address: Institut de Physique du Globe de Paris (IPGP), Université Paris Cité, Paris, France

Correction to: *Communications Chemistry* 10.1038/s42004-023-01075-1, published online 9 December 2023

In the version of the article initially published, in Fig. 3a, the label “Pristine SZ CVR=39% *d*=1.6 g cm^–3^” was incorrect and has now been amended to “Pristine SZ CVR=39% *d*=1.8 g cm^–3^”. Additionally, in the “Effect of ball-milling on the structure of SZ” section, the sentence “...higher than that of pristine SZ (1.6 g cm^−3^)” has been amended to “...higher than that of pristine SZ (1.8 g cm^−3^)”. These corrections have been made in the HTML and PDF versions of the article.

