# Supplementary material for: Siliceous zeolite-derived topology of amorphous silica
Source: Commun Chem. 2023 Dec 9;6:269. doi: 10.1038/s42004-023-01075-1 (PMC10710485; doi:10.1038/s42004-023-01075-1)
Supplement: Supplementary file 2 — Supplementary Information [file 42004_2023_1075_MOESM2_ESM.pdf]

Supplementary Materials for

Siliceous zeolite-derived topology of amorphous SiO_2

Hirokazu Masai*, Shinji Kohara*, Toru Wakihara, Yuki Shibazaki, Yohei Onodera, Atsunobu Masuno, Sohei Sukenaga, Koji Ohara, Yuki Sakai, Julian Haines, Claire Levelut, Philippe Hébert, Aude Isambert, David A. Keen, and Masaki Azuma

*Corresponding author. Email: hirokazu.masai@aist.go.jp and kohara.shinji@nims.go.jp

This PDF file includes:

Figures S1 to S5

Tables S1 to S2

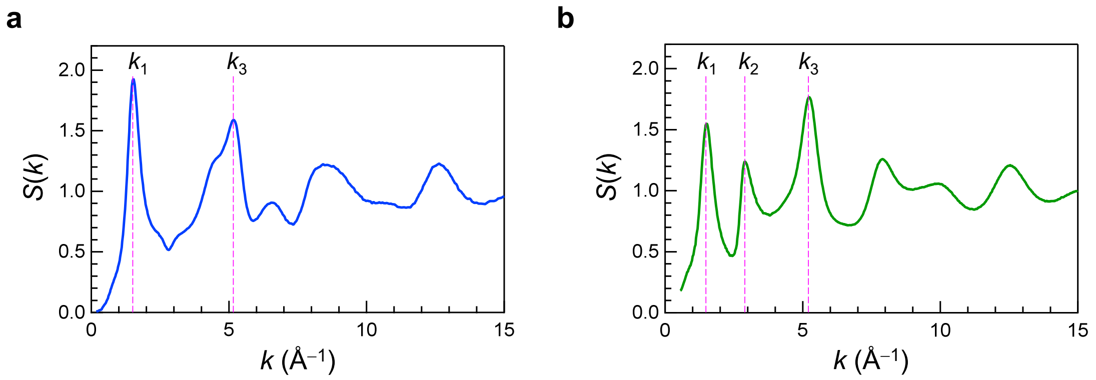

Figure S1 Comparison of k_1, k_2, and k_3 peaks in $S(k)$ of pristine glassy SiO_2 (GS)[19]. X-ray $S(k)$ (**a**) and Neutron $S(Q)$ (**b**) of pristine GS. The k_2 peak is observed only in Fig. S1b because k_2 reflects packing of oxygen atoms that (relatively) scatter neutrons more strongly than X-rays[17].

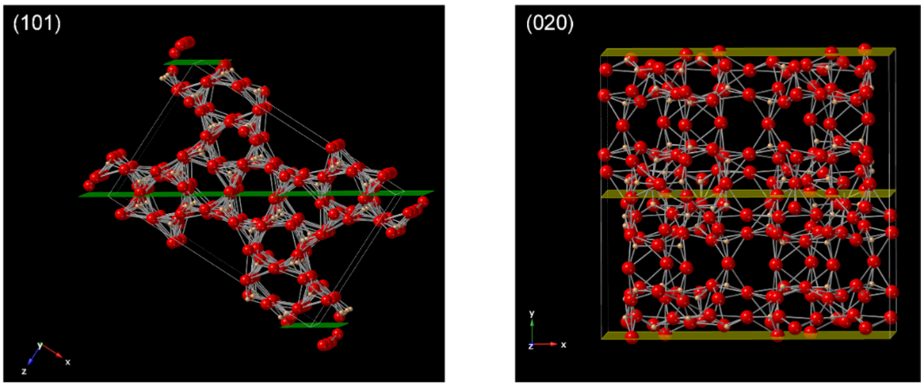

Figure S2 Illustration of siliceous zeolite (SZ) with highlight of (101) and (020) planes. The space group and crystal system are Pnma and orthorhombic, respectively. The *d*-spacings of (101) and (020) are 10.99789 and 9.86900 Å, respectively. (orange: silicon and red: oxygen).

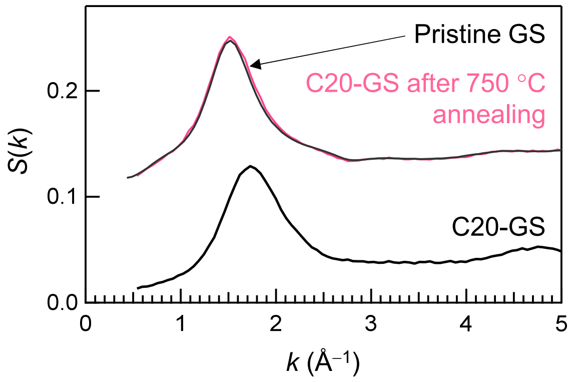

Figure S3 Comparison of $S(k)$ of densified glassy SiO_2 (GS) after cold compression with 20 GPa (C20-GS) before and after thermal annealing at 750 °C. After annealing, $S(k)$ of densified GS was recovered to be identical to that of pristine GS.

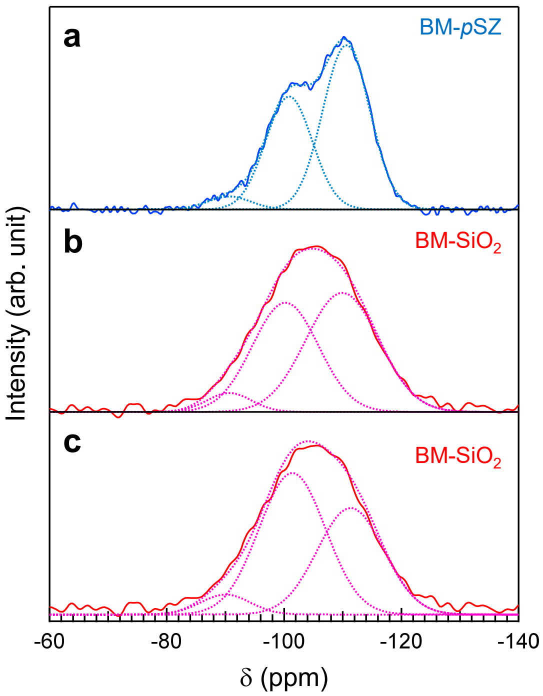

Figure S4 ^{29}Si MAS NMR spectra of powdered siliceous zeolite (SZ) (**a**) and SiO_2 **(b, c)** after BM-treatment. Three Gaussian curves were applied to reproduce the line shape of ^{29}Si NMR signals. Gaussian curves near –90, –100, and –110 ppm are assigned to Q^2, Q^3, and Q^4 species, respectively. The Q^2:Q^3:Q^4 ratios of SiO_2 after BM-treatment are 0.06:0.51:0.43 (**b**) and 0.06:0.54:0.40 (**c**) with different convolution parameters.

Supplementary reference

MacKenzie, K. J. D. & Smith, M. E., *Multinuclear solid-state NMR of inorganic materials*, pp. 205–208, Elsevier: Oxford, (2002).

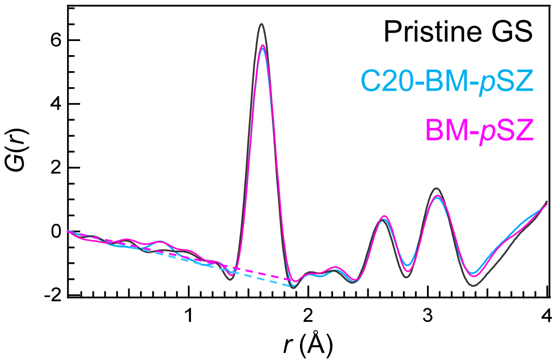

Figure S5 Enlarged $G(r)$ at low r region of Fig. 5e. (GS: Glassy SiO_2, BM-*p*SZ: Ball milled siliceous zeolite powder, C20-BM-*p*SZ: the densified amorphous SiO_2 obtained by cold compression of BM-*p*SZ with 20 GPa). The densities of ball-milled samples were calculated from the slope of dotted lines using $\rho = \frac{1}{4\pi}\frac{\partial G(r)}{\partial r}$.

Table S1 Fitting parameters of $G(r)$ in SiO_2-related materials. (SZ: siliceous zeolite, BM-*p*SZ: Ball milled SZ powder, and GS: Glassy SiO_2). Peak fitting was performed using a Gaussian function.

	Pristine SZ	BM-*p*SZ	Pristine GS[19]
Position (Å)	1.62	1.62	1.61
Half width half maximum (Å)	0.13	0.13	0.13
Height	8.27	6.99	8.01

Table S2 Fitting parameters of ^{29}Si MAS NMR spectra of siliceous zeolite powder (*p*SZ) before and after ball-milling (BM). Peak fitting was performed using a Gaussian function. Although Q^4 peak consists of different Q^4 species, two Q^4 species are used as the most simplified case.

		*p*SZ	BM-*p*SZ
Q^2	Position (ppm)		–91
	Half width half maximum (ppm)		5
	Height		0.1
Q^3	Position (ppm)	–103	–101
	Half width half maximum (ppm)	3	6
	Height	0.2	0.7
Q^4	Position (ppm)	–113	–111
	Half width half maximum (ppm)	2	6
	Height	0.9	0.9
Q^{4*}	Position (ppm)	–115	
	Half width half maximum (ppm)	2	
	Height	0.5	
